# The impact of storage protocols on the mechanical and structural properties of frozen platelet-rich fibrin membranes

**DOI:** 10.2340/biid.v12.44903

**Published:** 2025-10-29

**Authors:** Sruthima N.V.S. Gottumukkala, Rayapati Lois Sowmya Abhinandhitha, Gautami S. Penmetsa, Konathala S.V. Ramesh, Pasupuleti Mohan Kumar, Kanakamedala Anil Kumar, Bandaru Shanmukh

**Affiliations:** Department of Periodontics and Implantology, Vishnu Dental College, Vishnupur, Bhimavaram, West Godavari, Andhra Pradesh, India

**Keywords:** Elasticity, platelet-rich-fibrin, scanning electron microscopy, tensile strength

## Abstract

**Background & objectives:**

Platelet-rich fibrin (PRF) has shown promise in periodontal surgery and is found to promote bone regeneration. However, its limitations include low elasticity, short lifespan, and poor storage stability. This study aimed to overcome these limitations by developing frozen PRF and comparing the mechanical and structural properties of advanced-PRF (A-PRF) and two frozen PRF storage protocols.

**Methodology:**

This *in vitro* study used blood samples from 14 healthy volunteers to prepare A-PRF, which was stored at −20°C for 1 day (Fz-PRF1) or 7 days (Fz-PRF7). The study evaluated mechanical properties (tensile strength and elasticity) using a universal testing machine, and structural properties (Blood Elements Adhesion Index [BEAI] and fibrin density) using ***scanning electron microscopy*** (SEM) analysis. Results were analysed by Chi-square, independent t-tests, and Tukey post-hoc tests.

**Results:**

Comparing three PRF membranes, Fz-PRF7 showed significantly higher tensile strength than A-PRF and Fz-PRF1, and higher modulus of elasticity than A-PRF. However, SEM analysis revealed no significant differences in BEAI and fibrin density scores across groups.

**Conclusion:**

Fz-PRF7 showed improved mechanical properties, specifically tensile strength and elasticity, compared to A-PRF, overcoming some limitations of conventional PRF.

## Introduction

Periodontal regeneration, the ultimate goal of periodontal treatment, has undergone significant advancements in recent years [1, 2]. The exploration of various growth factors (GFs) has played a crucial role in this progress. In this context, platelet concentrates (PC) have emerged as a valuable tool in regenerative periodontal therapy due to their autologous origin [3]. These concentrates possess an exceptionally high concentration of platelets, GFs, and leukocytes, containing 90% of platelets and 50% of leukocytes compared to natural blood concentrations [3].

Platelet-rich fibrin (PRF), a fibrin meshwork made up of platelets, white blood cells, serum, and concentrated GFs, is one such product [4]. Advanced platelet-rich fibrin (A-PRF), which uses a low-speed centrifugation concept (LSCC) that applies lower G-forces, is the result of recent developments in PRF production. When compared to conventional PRF, this method produces a greater release of GFs [4, 5].

Platelet-derived growth factors (PDGF), transforming growth factor beta (TGF-β), and vascular endothelial growth factors (VEGF) are among the several autologous GFs that PRF offers in abundance and are crucial for bone regeneration. These GFs are released to surgical sites over an extended period of time [6–8]. Although PRF was first created as fresh platelet concentrates for same-day use in regenerative bone therapies [9, 10], its broad clinical application has been limited by its low elastic modulus, storage limitations, rapid resorption rate (usually within 2–3 weeks), and limitations in emergency therapy use [11, 12]. This fast breakdown limits its use as a barrier membrane, unlike collagen, which can exclude soft tissue growth over a longer period. However, recent studies show that applying thermal manipulation can extend PRF’s resorption timeframe from 2 to 3 weeks to over 4 months, and improve biologic properties [12–15].

Freezing has been shown to alter the structural, mechanical, and biological properties of PRF membranes. Frozen PRF (Fz-PRF), a modified preparation technique, may provide an autologous, stable, biocompatible membrane for tissue regeneration [14–16]. The use of autologous blood samples in regenerative therapy, such as platelet concentrates, necessitates on-site collection and immediate centrifugation for processing. However, there is a lack of established methods for processing and storage protocols. The ability to prepare and store PRF without significant reduction in bioactivity and improvement in mechanical properties would expand its clinical applications. Therefore, the current research hypothesised that Fz-PRF membranes stored for 1 day and 7 days would exhibit improved mechanical properties and altered structural properties compared to A-PRF membranes, with storage duration influencing the extent of preservation. This study aimed to evaluate the mechanical and structural properties of A-PRF and two Fz-PRF protocols: frozen for 1 day and frozen for 7 days.

## Methodology

### Study design and sample size

The study employed an experimental design to evaluate the mechanical and structural properties of A-PRF and two Fz-PRF protocols. The study used a within-subjects design, where blood samples from the same participants were used to prepare A-PRF and Fz-PRF protocols.

Calculations to determine the sample size was performed for difference in the mechanical and structural properties between different groups. The calculations were based on an effect size of 0.5, an alpha level of 0.05, and the desired power of 80%. The estimate sample size was 36. Considering 20% loss of follow up, 42 samples will be included in this study.

### Participant selection

A total of 14 systemically healthy student volunteers (8 males and 6 females) aged 20–25 years, who were non-smokers and not taking any medications in the past 3 months, participated in the study. The exclusion medications that can affect platelet concentrates include anti-inflammatory agents (NonSteroidal anti-inflammatory drugs (NSAIDs) and corticosteroids), antihistamines, and sedatives/hypnotics. In addition, beta blockers and ACE (angiotensin-converting enzyme) inhibitors may have indirect effects on platelet function through their cardiovascular effects; hence, excluded.

### Blood collection and platelet-rich fibrin preparation

Blood samples were drawn from each participant into three 10 ml tubes. Plain glass red top hydrophilic tubes were used for blood collection and A-PRF preparation. Informed consent was obtained from all participants prior to blood collection. For A-PRF preparation, blood samples were centrifuged at 1,300 rpm for 14 min using a fixed angle centrifuge (Dr Choukroun DUO Quattro PRF Centrifuge, Dental Implant technologies, United states). The A-PRF clots were extracted, separated from the red blood cell phase, and compressed for 5 min. For Fz-PRF, blood samples were centrifuged at 1,300 rpm for 14 min [4] and the obtained A-PRF clots were frozen at −20°C for 1 day (Fz-PRF1 Group/Test Group 1) or 7 days (Fz-PRF7 group/Test Group 2) under strict aseptic conditions.

### Scanning electron microscopy

**Figure 1:**
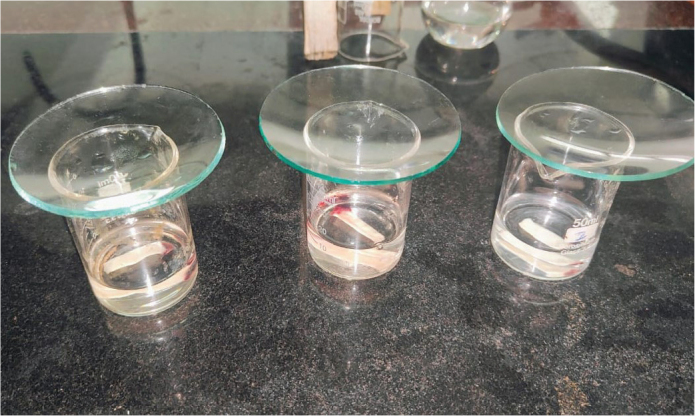
PRF membranes treated with 100% hexamethyldisilane for 10 min before processing for SEM analysis.

A-PRF membranes and also the two protocols of Fz-PRF, that is, A-PRF frozen at −20°C for 24 h/1 day (test group 1) or 7 days (test group 2) were fixed in 2.5% glutaraldehyde diluted in phosphate-buffered saline (PBS) for 4 h, followed by dehydration in increasing concentrations of ethanol (50%, 75%, 90%, and 100%) for 30 min each. The membranes were then treated with 100% hexamethyldisilane for 10 min and left on absorbent paper overnight in a laminar flow cabinet [17] (Figure 1).

After drying, the fresh A-PRF and also the two Fz-PRF membranes were coated with platinum, and the central region of each membrane was scanned using SEM. The thickness of the fibrin bundles and the density of the fibrin network were measured and compared between A-PRF and the two Fz-PRF protocols. Each membrane was scanned at magnifications of 5,000× and 10,000×, with three images taken at each magnification for fibrin thickness and density measurements.

### Fibrin network analysis

The thickness of fibrin bundles and the density of the fibrin network were measured and compared between A-PRF and Fz-PRF protocols. The fibrin network pattern was evaluated using the Blood Elements Adhesion Index (BEAI), which assigns scores ranging from 0 to 3. A score of 0 indicates the complete absence of fibrin network and blood cells, while a score of 1 signifies very sparse or scarcely distributed fibrin strands and/or blood cells. A score of 2 represents a moderate number of blood cells with thin fibrin network that shows poor interlacing, and a score of 3 denotes a dense fibrin network with rich interlacing accompanied by the presence of blood cells [18]. To objectively determine the density of fibres in the SEM images, ImageJ analysis software was used, involving image preprocessing, thresholding, and particle analysis to detect and measure fibre size and shape. The fibre density was then calculated by analysing the pore size between the fibrin network, allowing us to classify the SEM images as scarce, thin and dense based on the calculated porosity/density. The fibrin network can be classified as dense fibrin network with rich interlacing when porosity is below 15% (very compact), thin fibrin network with poor interlacing when porosity ranges between 15–35%, and very scarce fibrin network when porosity exceeds 35% [18, 19].

### Mechanical properties evaluation

To assess the tensile strength of A-PRF and two Fz-PRF protocols, tensile tests were conducted using a universal testing machine (Instron 5566). The samples, consisting of A-PRF, 1-day Fz-PRF, and 1-week Fz-PRF, were prepared and cut to a diameter of 10 mm and a length of 25 mm. The PRF dimensions were standardised or reduced to 10 mm x 25 mm to minimise inter-participant variability, ensuring a consistent size for comparison. Measurements were carefully recorded using a metallic ruler, placed alongside the PRF clot, to ensure accuracy and reproducibility. Slip-proof rubber sheets were used to keep the samples from slipping, and clamps were used to bind them firmly at both ends. The initial distance between the clamp faces was set to 5 mm for all samples. Tensile loading was applied at a crosshead speed of 10 mm/min, and the maximum load at specimen failure was recorded. Using the formula *S* = *F*/*A*, where F is the highest force (*N*) and A is the unit area (m2), the tensile strength was determined [20].

To evaluate the mechanical properties of A-PRF and two Fz-PRF protocols, nanoindentation tests were performed using a T1 950 Triboindenter (Hysitron Nanotechnology, Minneapolis, US). The modulus of elasticity and hardness of A-PRF, 1-day Fz-PRF, and 1-week Fz-PRF membranes were measured. A total of five readings from different locations on each membrane were recorded at a load of <1 μN, and the mean value was calculated.

### Statistical analysis

Data were entered into Microsoft Excel sheet and analysis was done using Statistical Package for the Social Sciences (SPSS) version 26.0 (IBM, Chicago). The results were analysed to compare the mechanical and structural properties of A-PRF and Fz-PRF protocols. Quantitative data were presented as mean and standard deviation, whereas qualitative data was presented as numbers and percentages. Results were analysed by Chi square, independent t tests and Tukey post-hoc tests. *P* ≤ 0.05 was considered statistically significant.

## Results

**Figure 2:**
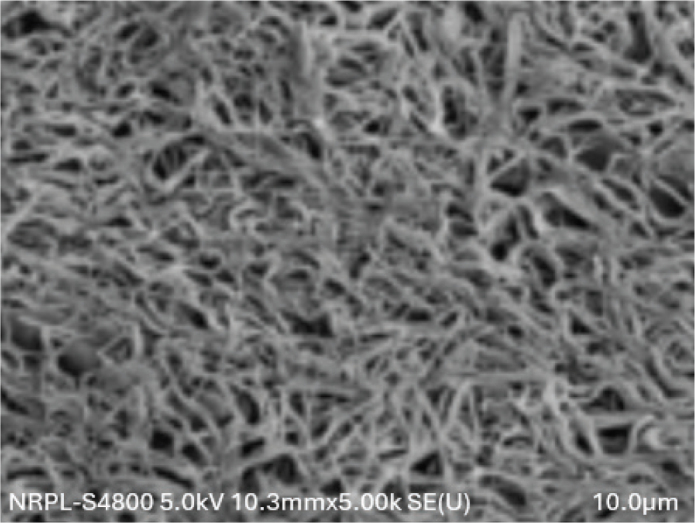
SEM image of control A-PRF showing fibrin network at 5.00K magnification.

**Figure 3:**
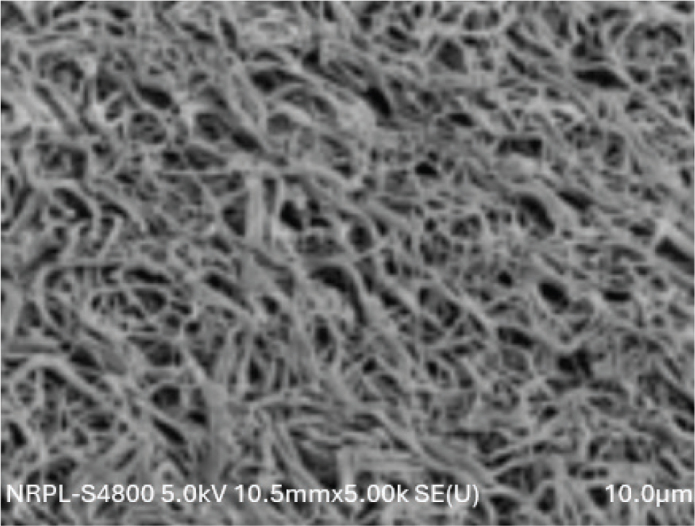
SEM image of test group Fz-PRF stored for 1 day showing fibrin network at 5.00K magnification.

**Figure 4:**
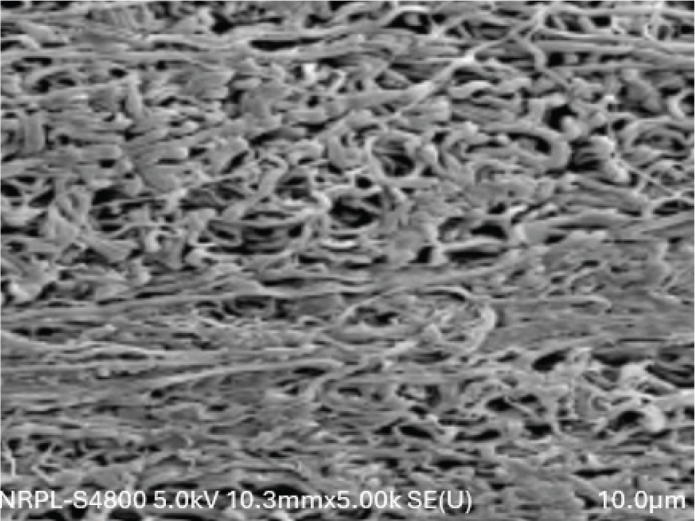
SEM image of test group Fz=PRF stored for 7 days showing fibrin network at 5.00K magnification.

The distribution of BEAI scores and the fibrin density pattern distribution were described from the SEM images, and are presented in Table 1 and Figures 2–4. All the three groups showed similar pattern of fibre distribution. Blood Elements Adhesion Index scores as assessed from the SEM images revealed, score 2, that is, thin fibrin network with poor interlacing, was the most frequent score in all three groups. Similarly, as regards the fibrin density pattern assessment using SEM images, dense fibrin network, was the most frequent score in all three groups. No significant differences were observed between the three groups for neither the BEAI scores (*p* = 0.896) nor the fibrin density scores (*p* = 0.896) (Figures 2–4).

**Table 1:** Score distribution according to BEAI and fibrin density analysis in control and test groups.

SEM evaluation	Score		Groups			Total	Chi square value	*P*
			A-PRF	Fz-PRF1	Fz-PRF7			
BEAI	1	Frequency	5	3	3	11	1.091	0.896
		% within Groups	35.7%	21.4%	21.4%	26.2%		
	2	Frequency	6	8	8	22		
		% within Groups	42.9%	57.1%	57.1%	52.4%		
	3	Frequency	3	3	3	9		
		% within Groups	21.4%	21.4%	21.4%	21.4%		
Fibrin density	Scarce	Frequency	6	8	8	22	1.091	0.896
		% within Groups	42.9%	57.1%	57.1%	52.4%		
	Thin	Frequency	5	3	3	11		
		% within Groups	35.7%	21.4%	21.4%	26.2%		
	Dense	Frequency	3	3	3	9		
		% of Total	21.4%	21.4%	21.4%	21.4%		

BEAI: Blood Elements Adhesion Index; Score 0: Complete absence of fibrin network and blood cells; Score 1: Very sparse or scarcely distributed fibrin strands and/or blood cells; Score 2: Moderate number of blood cells with thin fibrin network that shows poor interlacing; Score 3: Dense fibrin network with rich interlacing accompanied by the presence of blood cells; A-PRF: Advanced Platelet-rich-fibrin; Fz-PRF1: 1 day frozen PRF; Fz-PRF7: 7 days Frozen PRF.

Intergroup comparison of the mechanical properties showed that the tensile strength differed with statistical significance among the three groups (Table 2; *p* < 0.001). Similarly, statistically significant differences were found in modulus of elasticity (Table 2; *p* = 0.012).

**Table 2:** Tensile strength and modulus of elasticity of test and control group PRF membranes.

Properties	Groups	Mean (mPa)	95% confidence interval for mean		*F*	*P*
			Lower bound	Upper bound		
Tensile strength	A-PRF	0.265 ± 0.032	0.2461	0.2839	16.785	0.000
	Fz-PRF1	0.278 ± 0.036	0.2572	0.2999		
	Fz-PRF7	0.340 ± 0.040	0.3173	0.3641		
	Total	0.294 ± 0.049	0.2795	0.3100		
Modulus of Elasticity	A-PRF	119.585 ± 1.096	118.9525	120.2190	4.955	.012
	Fz-PRF1	121.985 ± 4.157	119.5853	124.3862		
	Fz-PRF7	124.207 ± 5.175	121.2187	127.1956		
	Total	121.926 ± 4.243	120.6039	123.2485		

A-PRF: Advanced Platelet-rich-fibrin; Fz-PRF1: 1 day frozen PRF; Fz-PRF7: 7 days Frozen PRF.

Post hoc Turkey tests found that Fz-PRF7 had significantly higher tensile strength than A-PRF and Fz-PRF1, while no difference was found between A-PRF and Fz-PRF1 (Table 3). As for modulus of elasticity, Fz-PRF7 had significantly higher modulus of elasticity than A-PRF, while there were no significant differences between A-PRF and Fz-PRF1 or between Fz-PRF7 and Fz-PRF1 (Table 3).

**Table 3:** Tukey’s post hoc test representing inter group comparison of Tensile strength and Young’s Modulus.

Property	Groups	Groups	Mean difference	Std. error	*P*	95% confidence interval	
						Lower bound	Upper bound
Tensile strength	A-PRF	Fz-PRF1	−0.01357	0.01394	0.598	−0.0475	0.0204
		Fz-PRF7	−0.07571	0.01394	0.000	−0.1097	−0.0418
	Fz-PRF1	Fz-PRF7	−0.06214	0.01394	0.000	−0.0961	−0.0282
Modulus of Elasticity	A-PRF	Fz-PRF1	−2.40000	1.46835	0.244	−5.9774	1.1774
		Fz-PRF7	−4.62143	1.46835	0.009	−8.1988	−1.0441
	Fz-PRF1	Fz-PRF7	−2.22143	1.46835	0.296	−5.7988	1.3559

A-PRF: Advanced Platelet-rich-fibrin; Fz-PRF1: 1 day frozen PRF; Fz-PRF7: 7 days Frozen PRF.

**Figure 5:**
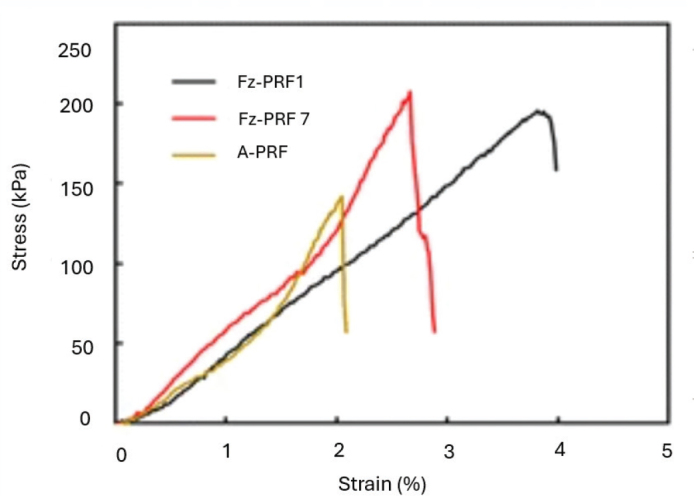
Stress strain curve of all the three PRF groups.

The stress strain curve shows that Fz-PRF7 exhibits the highest peak stress (~210 kPa) but fractures at a lower strain (~2.5–3%), reflecting greater stiffness but brittleness. In contrast, Fz-PRF1 withstands higher strain (~4%) with moderate peak stress, indicating greater elasticity and toughness, while A-PRF has lower strength and fails earlier (~2% strain) (Figure 5).

## Discussion

Platelet-rich fibrin is a biomaterial rich in GFs and platelets, promoting tissue regeneration and healing. However, its short shelf life and requirement for immediate use limit its clinical applications [21–23]. To overcome these limitations, researchers have explored freeze-drying as a method to enhance protein stability and preservation. Despite its potential, processing and lyophilising, PRF remains challenging as it is a complex procedure which cannot be performed for routine clinical care [24–26]. Therefore, developing a simple and effective clinical protocol with optimal mechanical and biological properties is crucial for advancing PRF applications. The present study aimed to evaluate the mechanical and structural properties of A-PRF and two Fz-PRF storage protocols, Fz-PRF1 and Fz-PRF7. The findings of this study demonstrated that Fz-PRF7 exhibited improved mechanical properties, specifically tensile strength and elasticity, compared to A-PRF and Fz-PRF1.

The tensile strength of Fz-PRF7 was significantly higher than A-PRF and Fz-PRF1, indicating that freezing PRF for 7 days enhances its resistance to deformation and failure. Similar findings were reported by Kardos et al. [27], where freeze-thawing enhanced tensile strength, cell adhesion, and durability of PRF membranes, making them suitable for use as surgical membranes. Their study used PRF membranes frozen at −20°C overnight, which showed a tensile strength of 0.25 ± 0.03, comparable to the Fz-PRF1 group in the current study (0.278 ± 0.036) [27]. This improvement in tensile strength can be attributed to the changes in the fibrin network structure and the increased cross-linking between fibrin fibres that occur during the freezing process. Similar mechanical properties for fresh PRF were observed by Khorshidi et al. with tensile strength of 0.20 ± 0.06 mPa [21].

The increased elasticity of Fz-PRF7, as measured by Young’s modulus, suggests that this protocol may provide a more stable and durable membrane for tissue regeneration. The control group’s tensile strength values were slightly lower than those reported by Ockerman et al., who found fresh PRF to have a tensile strength of 0.29 MPa. This discrepancy may be attributed to their use of L-PRF (Leukocyte-PRF) instead of A-PRF in the study [17].

In contrast to the mechanical properties, the structural properties evaluated using SEM showed no significant differences in BEAI scores and fibrin density scores among the three groups. This suggests that the freezing process does not significantly alter the fibrin network structure and the distribution of blood elements within the PRF membrane. Contrary to the results of the current study Kardos et al. observed that frozen PRF had a more rugged surface compared to the fresh PRF with more compact fibres with smaller pores between the fibres, compared to fresh membranes [27].

While the effects of freeze-thawing on platelet-rich plasma (PRP) have been extensively studied, showing either improved properties or minimal impact, there is limited research on the effects of freezing on PRF, highlighting a need for further investigation [17, 28]. The improved mechanical properties of Fz-PRF7 make it a promising protocol for clinical applications, particularly in periodontal regeneration and tissue engineering. The increased tensile strength and elasticity of Fz-PRF7 may provide a more stable and durable membrane for tissue regeneration, which can lead to improved clinical outcomes. However, our study indicates that freezing/thawing, and specifically Fz-PRF, does considerably enhance the mechanical characteristics and may be regarded as a storage option until clinical investigations investigate and explain the effects of PRF storage on patient complaints and functional improvement.

Further studies are needed to evaluate the biological properties of Fz-PRF7, such as its ability to promote cell proliferation and differentiation, and its potential for clinical applications. Additionally, the effects of freezing on the GF release and the stability of PRF membranes over time need to be investigated.

### Limitations and prospects

While this study provides valuable insights into the characterisation of PRF fibres, it has some limitations. The current study focused on SEM and ImageJ analysis, which provided a comprehensive understanding of fibre morphology and density. However, other characterisation techniques, such as Water Contact Angle, porosity testing, Fourier Transform Infrared spectroscopy (FTIR), and Differential Scanning Calorimetry (DSC), were beyond the scope of this study. Future studies could benefit from incorporating these additional characterization techniques to provide a more comprehensive understanding of PRF fibres investigating the hydrophilicity, porosity, chemical composition, and properties of PRF fibres, ultimately enhancing their potential applications in tissue engineering and regenerative medicine.

Also, the *in vitro* design may not accurately represent the complex oral environment, and the relatively small sample size of 14 participants may not be representative of the larger population. Additionally, the study only evaluated two short-term freezing protocols and a single freezing temperature. The mechanical testing was limited to tensile strength and elasticity, and the SEM analysis may not have captured all aspects of the structural properties of PRF membranes. Furthermore, the study did not evaluate the biological properties of PRF membranes, such as GF release and cell viability, which are crucial for clinical applications. Variability in PRF preparation techniques and the lack of long-term evaluation also limit the generalisability and applicability of the findings. To overcome these constraints and offer a more thorough comprehension of the characteristics and possible uses of frozen PRF membranes, additional research is required.

## Conclusion

The current study demonstrates that freezing PRF membranes for 7 days (Fz-PRF7) significantly improves their mechanical properties, specifically tensile strength and elasticity, compared to A-PRF. The structural properties, evaluated using SEM, showed no significant differences among the three groups. These findings suggest that Fz-PRF7 may provide a more stable and durable membrane for tissue regeneration, potentially leading to improved clinical outcomes.

## Clinical significance

The clinical significance of this study lies in its potential to improve the handling and durability of PRF membranes in periodontal regeneration and tissue engineering applications. The finding that freezing PRF membranes for 7 days enhances their mechanical properties, specifically tensile strength and elasticity, suggests that this protocol may provide a more stable and durable membrane for tissue regeneration. Additionally, the ability to store PRF membranes for longer periods without compromising their properties could increase their accessibility and convenience for clinicians, ultimately benefiting patients undergoing periodontal and regenerative treatments.

## Data Availability

All the data related to the current research is available in the manuscript. No additional information is required to be available in other sources.

## Permission to reproduce material from other sources

All the data provided in the article is original and not from any other sources.
